# Other PHAC publications

**DOI:** 10.24095/hpcdp.44.6.12

**Published:** 2024-06

**Researchers from the Public Health Agency of Canada also contribute to work published in other journals and books. Look for the following articles published in 2024:**

**Baddeliyanage R, Enns A, VanSteelandt A**, Abele B, Kouyoumdjian F, **Schleihauf E, Pan S, [...] Rotondo J**. Substance-related acute toxicity deaths by area-based characteristics: a descriptive analysis of a national chart review study of coroner and medical examiner data. Int J Ment Health Addiction. 2024. https://doi.org/10.1007/s11469-024-01259-3

Kelly SE, Benkhedda K, Brooks SPJ, MacFarlane AJ, **Greene-Finestone L**, Skidmore B, et al. Risk of bias in cross-sectional studies: protocol for a scoping review of concepts and tools. MethodsX. 2024;12. https://doi.org/10.1016/j.mex.2024.102610

**Khan F**, Coutts SB, Hill MD. Letter by Khan et al. regarding article, “Long-term incidence of ischemic stroke after transient ischemic attack: a nationwide study from 2014 to 2020.” Circulation. 2024;149(10):797-8. https://doi.org/10.1161/CIRCULATIONAHA.123.067840

Lange S, Kim KV, Lasserre AM, **Orpana H**, Bagge C, Roerecke M, et al. Sex-specific association of alcohol use disorder with suicide mortality: a systematic review and meta-analysis. JAMA Netw Open. 2024:E241941. https://doi.org/10.1001/jamanetworkopen.2024.1941

Lozano D, Dohoo C, Elfstrom D, Carswell K, **Guthrie JL**. COVID-19 outbreak at a residential apartment building in Northern Ontario, Canada. Epidemiol Infect. 2024;152:e53. https://doi.org/10.1017/S0950268824000256

MacNeil A, Li G, Gulati I, Taunque A, **Jiang Y, de Groh M**, et al. Depression during the COVID-19 pandemic among older adults with stroke history: findings from the Canadian Longitudinal Study on Aging. Int J Geriatr Psychiatry. 2024;39(2):e6062. https://doi.org/10.1002/gps.6062

Peng A, Bosco S, Simmons AE, **Tuite AR**, Fisman DN. Impact of community mask mandates on SARS-CoV-2 transmission in Ontario after adjustment for differential testing by age and sex. PNAS Nexus. 2024;3(2):pgae065. https://doi.org/10.1093/pnasnexus/pgae065

**Rolland-Harris E, Bryan S**, VanTil L. Studying military and veteran health using a life-course approach: lessons learned from a Canadian record linkage study. J Mil Veteran Fam Health. 2024;10(1):7-15. https://doi.org/10.3138/jmvfh-2022-0071

**Saxena S, Zutrauen S, McFaull SR**. Assault-related traumatic brain injury hospitalizations in Canada from 2010 to 2021: rates, trends and comorbidity. Inj Epidemiol. 2024;11(1). https://doi.org/10.1186/s40621-024-00486-5

Warkentin MT, Ruan Y, Ellison LF, Billette JM, **Demers A**, Liu F, et al. Progress in site-specific cancer mortality in Canada over the last 70 years. Sci Rep. 2024;14(1):5688. https://doi.org/10.1038/s41598-024-56150-x

